# Abnormal sleep physiology in children with 15q11.2-13.1 duplication (Dup15q) syndrome

**DOI:** 10.1186/s13229-021-00460-8

**Published:** 2021-08-03

**Authors:** Vidya Saravanapandian, Divya Nadkarni, Sheng-Hsiou Hsu, Shaun A. Hussain, Kiran Maski, Peyman Golshani, Christopher S. Colwell, Saravanavel Balasubramanian, Amos Dixon, Daniel H. Geschwind, Shafali S. Jeste

**Affiliations:** 1 Center for Autism Research and Treatment, Semel Institute for Neuroscience, University of California, Los Angeles, Los Angeles, CA 90024 USA; 2 Neuroscience Interdepartmental Ph.D. Program, University of California, Los Angeles, Los Angeles, CA 90095 USA; 3 Division of Pediatric Epilepsy, Department of Pediatric Neurology, Children’s Hospital Medical Center of Akron, Akron, OH 44308 USA; 4 Swartz Center for Computational Neuroscience, UC San Diego, La Jolla, USA; 5 Division of Pediatric Neurology, David Geffen School of Medicine, UCLA Mattel Children’s Hospital, Los Angeles, CA USA; 6 Department of Neurology, Boston Children’s Hospital and Harvard Medical School, Boston, MA USA; 7 Department of Neurology and Semel Institute for Neuroscience, David Geffen School of Medicine, 710 Westwood Plaza, Los Angeles, CA 90095 USA; 8 West Los Angeles VA Medical Center, 11301 Wilshire Blvd, Los Angeles, CA 90073 USA; 9 Department of Psychiatry and Biobehavioral Sciences, Semel Institute, University of California, Los Angeles, Los Angeles, CA 90095 USA; 10 Microsoft, Sunnyvale, CA 94089 USA; 11 Undergraduate Interdepartmental Program for Neuroscience, University of California, Los Angeles, Los Angeles, CA 90095 USA; 12 Children’s Hospital Los Angeles, Los Angeles, CA USA

**Keywords:** Dup15q syndrome, Autism, Sleep, EEG, GABA_A_R, UBE3A, Spindles, Slow wave sleep, Biomarkers

## Abstract

**Background:**

Sleep disturbances in autism spectrum disorder (ASD) represent a common and vexing comorbidity. Clinical heterogeneity amongst these warrants studies of the mechanisms associated with specific genetic etiologies. Duplications of 15q11.2-13.1 (Dup15q syndrome) are highly penetrant for neurodevelopmental disorders (NDDs) such as intellectual disability and ASD, as well as sleep disturbances. Genes in the 15q region, particularly *UBE3A* and a cluster of GABA_A_ receptor genes, are critical for neural development, synaptic protein synthesis and degradation, and inhibitory neurotransmission. During awake electroencephalography (EEG), children with Dup15q syndrome demonstrate increased beta band oscillations (12–30 Hz) that likely reflect aberrant GABAergic neurotransmission. Healthy sleep rhythms, necessary for robust cognitive development, are also highly dependent on GABAergic neurotransmission. We therefore hypothesized that sleep physiology would be abnormal in children with Dup15q syndrome.

**Methods:**

To test the hypothesis that elevated beta oscillations persist in sleep in Dup15q syndrome and that NREM sleep rhythms would be disrupted, we computed: (1) beta power, (2) spindle density, and (3) percentage of slow-wave sleep (SWS) in overnight sleep EEG recordings from a cohort of children with Dup15q syndrome (*n* = 15) and compared them to age-matched neurotypical children (*n* = 12).

**Results:**

Children with Dup15q syndrome showed abnormal sleep physiology with elevated beta power, reduced spindle density, and reduced or absent SWS compared to age-matched neurotypical controls.

**Limitations:**

This study relied on clinical EEG where sleep staging was not available. However, considering that clinical polysomnograms are challenging to collect in this population, the ability to quantify these biomarkers on clinical EEG—routinely ordered for epilepsy monitoring—opens the door for larger-scale studies. While comparable to other human studies in rare genetic disorders, a larger sample would allow for examination of the role of seizure severity, medications, and developmental age that may impact sleep physiology.

**Conclusions:**

We have identified three quantitative EEG biomarkers of sleep disruption in Dup15q syndrome, a genetic condition highly penetrant for ASD. Insights from this study not only promote a greater mechanistic understanding of the pathophysiology defining Dup15q syndrome, but also lay the foundation for studies that investigate the association between sleep and cognition. Abnormal sleep physiology may undermine healthy cognitive development and may serve as a quantifiable and modifiable target for behavioral and pharmacological interventions.

**Supplementary Information:**

The online version contains supplementary material available at 10.1186/s13229-021-00460-8.

## Background

Neurodevelopmental disorders (NDDs), such as autism spectrum disorders (ASD), intellectual disability (ID) and attention deficit-hyperactivity disorder (ADHD), affect 1–2% of the general population. Sleep problems are highly prevalent in NDDs [1, 2], with 50–95% of children meeting criteria for a sleep disorder at a behavioral level [3–5]. Studies have estimated that sleep disturbances occur in 40–80% of children with ASD [6–9], with poor sleep being associated with greater autism severity, cognitive impairment and behavioral challenges [10, 11]. Healthy sleep physiology plays an essential role in overall health and cognitive development [12–17], and sleep micro- and macrostructures—particularly spindles and slow wave sleep (SWS)—are critical for learning, memory consolidation and overall intellectual ability [18–22]. Abnormal spindle number and morphology are associated with epilepsy, as well as with neurodevelopmental and neuropsychiatric disorders [23–28]. Deficits in SWS have been reported in genetic forms of ASD and NDDs such as Rett syndrome [29, 30], as well as in non-syndromic ASD [31]. Specific sleep microarchitecture alterations in ASD, however, have been inconsistent likely as a result of the heterogeneity of the condition and differences in analytic techniques.

Maternally derived duplications of chromosome 15q11.2-13.1 collectively represent one of the most common copy number variants associated with NDDs [32, 33] and result in a clinical syndrome (Dup15q syndrome) that includes delays across developmental domains, high penetrance for ASD, and epilepsy [34–37]. Two primary duplication types result in the syndrome: (1.) an interstitial duplication or trisomy, resulting in one extra copy of the 15q region that lies on the same chromosome arm, or (2.) an isodicentric duplication, resulting in two extra copies of the region on a supernumerary chromosome [35]. Several genes within the 15q critical region are overexpressed in Dup15q syndrome, notably: (1.) *UBE3A*, a gene that encodes a ubiquitin protein ligase which is imprinted in neurons [38, 39] and regulates synaptic development and function [40–43], and (2.) a cluster of gamma-aminobutyric acid type A receptor (GABA_A_R) genes, *GABRB3*, *GABRA5*, and *GABRG3,* which encode the β3, α5 and γ3 receptor subunits, respectively. Several studies have shown that both in humans and in mouse models, mutations in the GABA_A_R genes result in autism and epilepsy phenotypes [44–48]. Loss of neuronal expression of the maternally inherited *UBE3A* gene due to deletions of the 15q critical region results in Angelman Syndrome (AS) [49, 50], which has some clinical overlap with Dup15q syndrome namely ID, ASD, and epilepsy.

On awake electroencephalography (EEG), children with Dup15q syndrome generate a prominent electrographic biomarker, defined by increased beta band oscillations (12–30 Hz), which distinguishes them from typically developing children as well as from those with non-syndromic ASD [36, 51, 52]. This EEG signature resembles the pattern seen in patients taking benzodiazepines or other positive allosteric modulators of GABA_A_ receptors, suggesting that this biomarker reflects aberrant GABAergic neurotransmission [53–55]. As a follow-up to the quantification of these abnormal awake EEG oscillations, we asked whether sleep physiology was also affected in Dup15q syndrome. We quantified the following metrics from overnight clinical EEG and polysomnogram (PSG) recordings: (1) beta band oscillations, (2) spindle density and (3) percentage of SWS, and compared them with age-matched neurotypical (NT) controls. Disruptions in these sleep rhythms can significantly affect overall quality of life and functionality, while exacerbating the severity of existing developmental and cognitive problems associated with NDDs. We hypothesized that sleep physiology—including beta power in sleep, sleep spindles, and SWS—would be abnormal in children with Dup15q syndrome. Findings from this study could lay the foundation for future investigation of the relationship between sleep EEG and cognition and the identification of potential pharmacological targets to improve not only sleep, but overall neurodevelopmental outcomes, in this syndrome.

## Methods

### Study participants

The study consisted of 27 participants. Overnight clinical EEG data on 25 participants were collected at the University of California, Los Angeles (UCLA) Ronald Reagan Medical Center, and overnight PSGs were collected from 2 participants at the Boston Children’s Hospital (BCH). All data were retrospectively identified. Fifteen recordings—including 13 clinical EEGs and EEG data extracted from 2 PSGs—were obtained from children with Dup15q syndrome, ranging in age from 9 months to 13 years. Twelve clinical EEGs were from age-matched NT children, ranging in age from 7 months to 14 years. The ages of the children in the two groups did not significantly differ, averaging 5.69 years in Dup15q syndrome and 5.78 years in NT controls. Each child with Dup15q syndrome had a confirmed genetic diagnosis of the syndrome and was either clinically referred through the Dup15q clinic at UCLA or recruited through the UCLA Intellectual Disability and Development Research Center (IDDRC). Participants with Dup15q syndrome from BCH were referred by a healthcare provider to the BCH Pediatric Sleep Laboratory to evaluate clinical concerns for restless sleep, sleep disordered breathing or periodic limb movements of sleep. Details of age, sex, duplication type, epilepsy status, frequency of spikes and medications for all children with Dup15q syndrome in the study can be found in Table 1.

**Table 1:** Dup15q syndrome participant characteristics

Age (months)	Gender	Duplication type	Epilepsy (active)	Spike-wave index in sleep	Medications (generic)
105	Female	Isodicentric	No	< 35%	Risperidone Melatonin
23	Female	Isodicentric	No	< 35%	None
108	Female	Isodicentric	No	< 35%	None
18	Male	Interstitial	No	< 35%	None
35	Male	Isodicentric	No	< 35%	None
54	Male	Isodicentric	No	< 35%	None
68	Female	Isodicentric	Yes	45–50%	Clobazam Topiramate
137	Male	Isodicentric	Yes	40–45%	Topiramate
73	Female	Interstitial	Yes	< 35%	Lamotrigine Guanfacine
19	Male	Isodicentric	Yes	35–40%	Vigabatrin Prednisolone
57	Female	Isodicentric	Yes	< 35%	None
9	Female	Isodicentric	Yes	< 35%	Levetiracetam Phenobarbital
55	Male	Isodicentric	Yes	65–70%	None
108	Male	Isodicentric	Yes	< 35%	None
156	Male	Isodicentric	Yes	40–45%	None

This table describes the characteristics of participants in the Dup15q syndrome cohort. Details on age, gender, epilepsy status and medications were extracted from participant background questionnaires, and duplication type was extracted from participant genetic reports. The percentage of sleep occupied by spike-waves was reported as the spike-wave index in clinical EEG reports and was verified by a board-certified pediatric epileptologist

Dosages were not available for all the medications listed, hence not included in the table

Based on direct assessments using the Autism Diagnostic Observation Schedule (ADOS), obtained from a prior study [56], or on clinical reports of previously conducted assessments, all 15 Dup15q syndrome participants in this study met criteria for ASD. The NT control group included children that were admitted to UCLA for EEG evaluation of paroxysmal events or spells that were ultimately determined to be non-epileptic in the context of normal EEG results. These participants had no historical or contemporaneous diagnoses of developmental delay, epilepsy, or other neurological disorders.

### EEG data acquisition

All overnight EEG data were retrospectively identified in accordance with the Institutional Review Board. EEGs from UCLA were acquired from the Pediatric Neurophysiology Laboratory at the UCLA Ronald Reagan Medical Center and were recorded at a sampling rate of 200 Hz, utilizing a standard 10–20 montage, 21 channel gold disc electrode placement recording set up on a Neurofax Polysmith DMS 11.0 Build 8093 with 921 amplifiers (Nihon Kohden America Inc, Irvine, CA). Data from BCH were recorded at a sampling rate of 200 Hz using XLTEK PSG system and Natus SleepWorks software (Natus Medical Inc., San Carlos, CA). Data were extracted and converted into European Data Format (EDF) for analysis.

### EEG data processing and analysis

Overnight EEG data were reviewed for timestamps, and the recording between approximately 10 p.m. and 5 a.m. was extracted. In the absence of formal sleep staging on clinical EEGs, this window was selected in order to maintain a comparable duration of potential sleep epochs between the two groups. Therefore, about 7 h of continuous overnight EEG recording were included for all participants, and the duration of the recording was not significantly different between the two groups (Dup15q, 7.03 h vs. NT, 7.06 h).

Raw EEG data were processed using the EEGLAB [57] software toolbox for MATLAB. Data were high-pass filtered at 1.0 Hz and low-pass filtered at 50 Hz with zero-phase FIR filters and forward–backward filtering. EEG channels with poor signal quality were automatically removed and interpolated with the following criteria: (1) spectral power between 1 and 50 Hz that was three standard deviations above or below that of other channels, (2) channels with flat signals (i.e. zeros) longer than 5 s, (3) channels that were poorly correlated (*r* < 0.7) with their reconstructed versions based on adjacent channels, (4) channels with line noise power four standard deviations higher than their signals, using *clean_rawdata()* function in EEGLAB. The interpolated EEG data were then re-referenced to the common average reference.

The power line noise (i.e. 60 Hz) was further removed using CleanLine in EEGLAB [58]. Artifact subspace reconstruction (ASR) was applied using *clean_asr()* function (*σ* = 20) [59] to automatically remove and interpolate non-stationary, high-amplitude bursts such as eye blinks, eye movement activity, possible complex epileptiform activity as well as motion artifacts. Independent component analysis (ICA) was performed, and an automatic independent components (IC) classifier, ICLabel [60], was used to separate and label ICs into seven categories. The ICs labeled as muscle, eye, heart, line noise, and channel noise with probability higher than 0.5 were rejected. Considering the performance variability in applying ICLabel to children and infant EEG versus adult EEG [61], the ICLabel output was visually inspected and reviewed. The final cleaned channel signals were reconstructed using the remaining ICs. Time–frequency analysis was performed for each channel of the cleaned overnight EEG using *spectrogram()* function in MATLAB with a Hanning window of 60-s and a 30-s overlap. The mean power at beta (12–30 Hz) and delta (1–4 Hz) band oscillations was further obtained for each epoch.

Raw EEG data were also manually reviewed by a board-certified pediatric epileptologist who was blind to diagnosis and group status. Artifacts, including eye blinks, eye movements, muscle, movement, electrocardiogram, and electrode artifacts, were visually identified. Sleep staging was performed on all clinical EEGs based on scoring criteria from the American Academy of Sleep Medicine (AASM) and was verified on PSG data in order to distinguish between wakefulness, non-rapid eye movement (NREM) sleep and rapid eye movement (REM) sleep. A visual overview of sleep stages and normal sleep architecture can be found in Additional file 1 and Additional file 2. Non-rapid eye movement (NREM) sleep cycles are categorized into stages including: N1, the lightest stage of sleep characterized by a dropout of eye, muscle and movement artifacts, as well as emergence of low-amplitude mixed frequency activity and vertex waves; N2, a deeper sleep stage characterized by the presence of sleep spindles and K-complexes; and N3 or slow-wave sleep (SWS), the deepest stage of sleep, characterized by the presence of slower frequencies (0.5–2 Hz) and high-amplitude signals. REM sleep, a stage characterized by vivid dreaming, is defined by mixed frequency brain wave activity similar to that seen during wakefulness with overriding eye movement artifact. REM sleep was not evaluated in this study given that electrooculogram (EOG) leads are not placed by default when recording clinical EEGs, thus limiting our ability to definitively score this sleep stage according to AASM criteria. The awake state was determined by the presence of eye, muscle and movement artifacts. Total wake and sleep time were aggregated but not independently analyzed given that not all arousals from sleep are enumerated and staged as wakefulness per AASM criteria, therefore hindering our ability to calculate these parameters with precision.

### Spindle detection

Sleep spindles were quantified and visualized using YASA (Yet Another Spindle Algorithm), a Python-based toolbox for automated multi-channel spindle detection [62]. Spectral power in the spindle frequency range (11–16 Hz) was first obtained relative to the total power in the broadband frequency (1–30 Hz) for all channels, using a 2-s window with a 200-ms overlap. Only the windows in which 20% of the signals’ total power was contained within the spindle frequency range were kept, in order to avoid false detection due to artifacts [62]. The selected windows of spindle activity were then reviewed for morphological features. Spindles that were less than 500 ms apart were merged, and those that were < 0.5 s or > 2.0 s in duration were eliminated. In order to avoid double counting, spindles detected with an initiation interval of < 300 ms were considered to be a single event. Throughout the hours of 10 p.m. and 5 a.m. across study participants, spindles were identified and quantified in all artifact-free sleep epochs with durations of at least 2 min. Spindle density was calculated as the number of spindles per minute for each epoch and averaged across epochs for each subject.

Spindles were also quantified manually. Similar to the automated method, spindles were visually identified and quantified in all artifact-free sleep epochs with durations of at least 2 min throughout the same hours of 10 pm and 5 a.m. Spindle density was calculated as the number of spindles per minute for each epoch and averaged across epochs for each subject.

### Slow-wave sleep analysis

Delta power (1–4 Hz) was computed for every 30-s epoch of the cleaned overnight EEG. SWS was automatically identified as epochs with higher delta power relative to non-SWS periods. Specifically, the non-SWS periods were identified as the 50% of all 30-s epochs in the sleep recording with the lowest delta power. The mean and standard deviation of the delta power over the non-SWS periods were computed. For the whole sleep recording, “high-delta” epochs were identified in which delta power was higher than one standard deviation above the mean non-SWS delta power. To avoid false positives (i.e., from sporadic motion artifacts), the final SWS periods excluded all the epochs with less than 32 consecutive high-delta epochs (i.e., 16 min) with methods based on a prior study [29]. The percentage of the amount of time spent in high-delta epochs across the 7 h of overnight EEG recording was defined as percent SWS for the study.

SWS also was manually quantified based on scoring criteria from the AASM, defined by 0.5–2 Hz slow waves of at least 75 μV occupying at least 20% of consecutive 30-s epochs [63]. The duration of each N3 sleep epoch was calculated across the 7 h of overnight EEG. Percentage of SWS was calculated as the total amount of time each subject spent in N3 sleep as a function of total sleep time.

All statistical analyses were performed using GraphPad Prism 8 software. Student’s *t* tests were used to compare spectral power, spindle density and percentage of SWS between groups. In all the figures, the asterisk indicates *p* < 0.05, **p < 0.01, ***p < 0.001 and ****p < 0.0001.

## Results

### Manual evaluation of sleep architecture

**Fig. 1:**
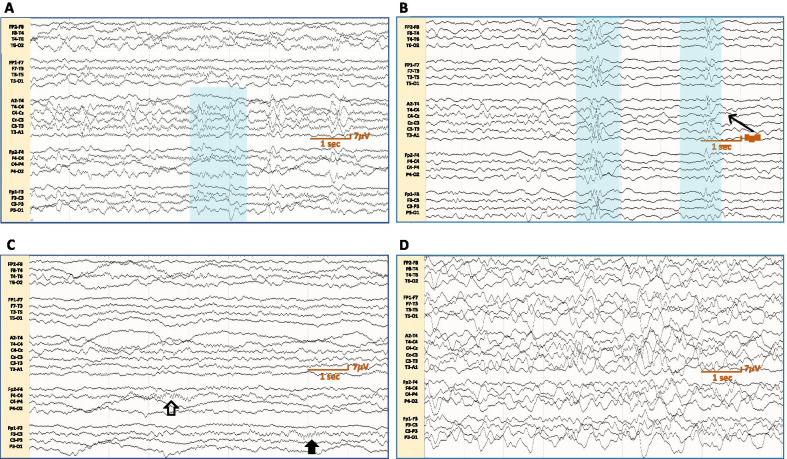
Sleep stages (N1 to N3) in children with Dup15q syndrome. Representative 9-s traces of continuous sleep EEG recording from children with Dup15q syndrome depicting vertex waves (field highlighted by blue rectangle) during stage N1 in a 35-month-old patient (**A**), K-complexes (broad field highlighted by blue rectangles) during stage N2 juxtaposed with sleep spindles (arrow) in a 19-month-old patient (**B**), asynchronous spindles in the right (hollow arrow) and left (solid arrow) frontocentral electrodes during stage N2 in a 54-month-old patient (**C**) and slow-wave sleep during stage N3 in a 35-month-old patient (**D**)

Manual staging of sleep EEGs indicated that all children with Dup15q syndrome demonstrated progression through NREM sleep cycles N1 and N2. Excessive beta oscillations were identified throughout the sleep recordings in all children with Dup15q syndrome but fluctuated between sleep stages, notably with a qualitative drop in stage N2. Vertex waves were observed in stages N1 and N2 (Fig. 1A). Sleep spindles and K-complexes emerged in stage N2 (Fig. 1B). Two children with Dup15q syndrome, aged 54 months and 156 months, demonstrated markedly abnormal sleep spindles—the former with frequent hemispheric asynchrony of sleep spindles (Fig. 1C), and the latter with poor spindle morphology and attenuated spindle voltages. However, not all children entered stage N3, as they did not meet AASM frequency criteria for slow waves. Of those who achieved SWS (Fig. 1D), half demonstrated fewer N3 cycles and reduced aggregate duration of N3 compared to NT controls.

Epileptiform activity was noted in 9 children with Dup15q syndrome and in all children with dual diagnoses of Dup15q syndrome and epilepsy. Observed epileptiform activity varied widely between participants and included generalized spike-wave discharges; focal or multifocal spikes, sharp waves and spike-waves; and focal or generalized paroxysmal fast activity.

### Beta oscillations in sleep

**Fig. 2:**
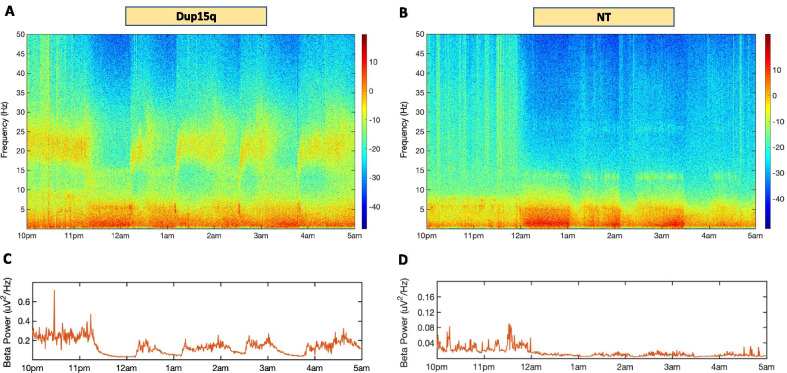
Persistent overnight beta oscillations in Dup15q syndrome. Time–frequency plot derived from 7 h of overnight sleep EEG from a 19-month-old representative Dup15q syndrome participant (**A**) and a 19-month-old representative neurotypical (NT) participant (**B**). Beta power (absolute power) dynamics plotted across the night in the 19-month-old participant with Dup15q syndrome (**C**) and in the 19-month-old NT participant (**D**)

Time–frequency analysis of the overnight EEG recordings revealed that children with Dup15q syndrome had visible and quantifiable beta oscillations (12–30 Hz) throughout sleep. Figure 2 shows examples of time–frequency plots from a child with Dup15q syndrome (Fig. 2A) and a child in the NT control group (Fig. 2B). Beta power was much higher and changed over time in the EEG recording from the child with Dup15q syndrome (Fig. 2C) compared to the child in the NT control group (Fig. 2D). Additional representative plots from participants with Dup15q syndrome are included in Additional file 3A, B.

**Fig. 3:**
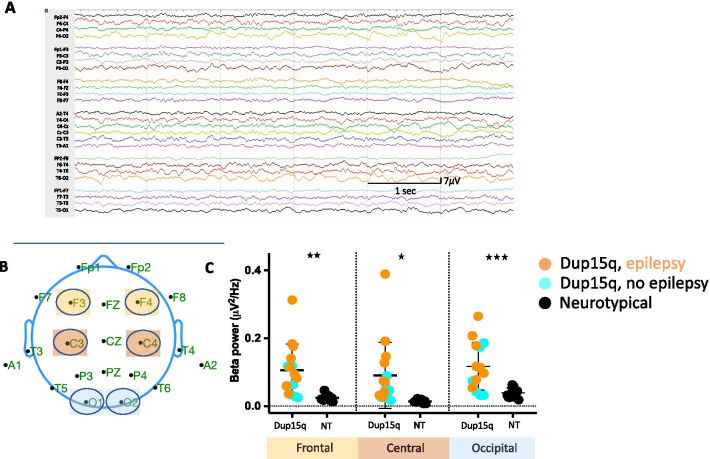
Elevated beta power in sleep in children with Dup15q syndrome. 6 s of continuous sleep EEG recording from a 19-month-old Dup15q participant (**A**). A scalp map showing standard 10–20 EEG electrode placements on the scalp, with channel groups of interest highlighted (frontal: yellow, central: red and occipital: blue) (**B**). Dot plots of absolute beta power (12–30 Hz) averaged across overnight sleep EEG, in the Dup15q syndrome group (turquoise: participants with no epilepsy, orange: participants with epilepsy) and the NT group (black), plotted for each channel group (**C**)

Mean beta power calculated across the overnight recording was significantly different between the groups. Although variable across individuals, in all three spatial locations (Fig. 3B), beta power was significantly higher (frontal: *p* = 0.001; central: *p* = 0.01; occipital: *p* = 0.0009) in children with Dup15q syndrome compared to age-matched NT controls (Fig. 3C). Within the Dup15q syndrome cohort, there were no differences in beta power based on the presence of epilepsy or between duplication types.

### Spindles

**Fig. 4:**
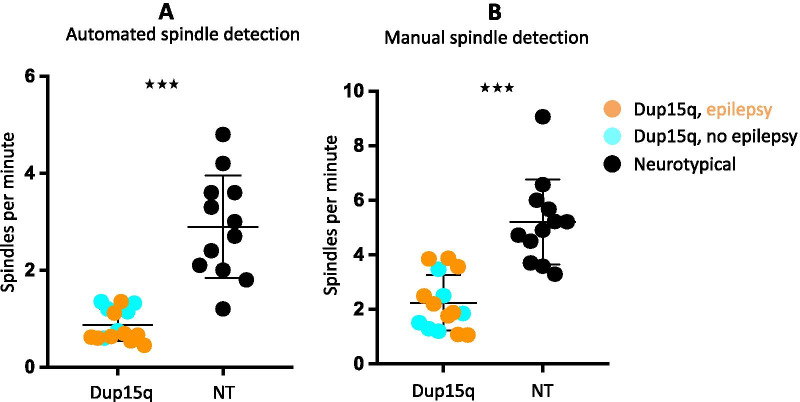
Reduced sleep spindle density in children with Dup15q syndrome. Dot plots of average spindle density in participants in the Dup15q syndrome group (turquoise: participants with no epilepsy, orange: participants with epilepsy) and the NT group (black), using automated spindle detection (**A**) and manual spindle detection (**B**) methods

Both the automated spindle detection algorithm and manual spindle detection revealed that children with Dup15q syndrome had significantly fewer spindles (*p* < 0.0001) compared to age-matched NT controls (Fig. 4A, B). Spindle density did not correlate with age, and there were no differences in duration or amplitude of spindles between groups. Within the Dup15q syndrome cohort, there were no significant differences in spindle density based on the presence of epilepsy or between duplication types.

### SWS

**Fig. 5:**
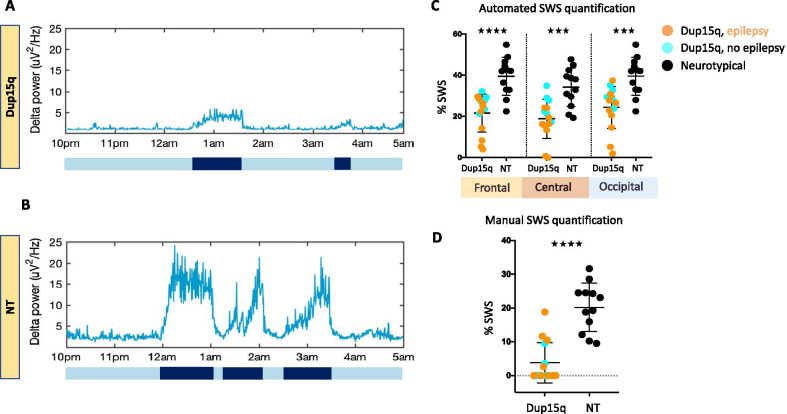
Reduced SWS in children with Dup15q syndrome. Delta (1–4 Hz) power dynamics across 7 h of overnight EEG from a 19-month-old Dup15q syndrome participant (**A**) and a 19-month-old NT participant (**B**), scored for high delta cycles (black) and low delta cycles (blue). Dot plots of percentage of SWS in participants in the Dup15q syndrome group (turquoise: participants with no epilepsy, orange: participants with epilepsy) and the NT group (black), using automated SWS quantification (**C**) and manual SWS quantification (**D**) methods. Different channel groups are highlighted in different colors (frontal: yellow, central: red and occipital: blue)

Quantitative analysis of SWS revealed markedly reduced SWS in children with Dup15q syndrome. There were significant group differences in the time spent in each discrete segment of SWS, the total amount of time spent in high delta cycles (Fig. 5A) and the percentage of SWS in all three channel groups (frontal, *p* < 0.0001; central, *p* = 0.0003; occipital, *p* = 0.0005), based on automated SWS detection. These differences indicated that children with Dup15q syndrome spent significantly less time in SWS compared to age-matched NT controls (Fig. 5B).

Manual quantification of SWS was consistent with automated SWS detection, revealing that 9 out of 15 children within the Dup15q syndrome group did not demonstrate N3 SWS and that children with Dup15q syndrome showed significantly less time in SWS (*p* < 0.0001) compared to the NT controls (Fig. 5C). Within the Dup15q syndrome cohort, there was no difference in the percentage of SWS based on the presence of epilepsy or between duplication types.

## Discussion

In this study, we quantified parameters of sleep physiology in children with duplications of 15q11.2-13.1, a genetic NDD highly penetrant for ASD, and compared them to age-matched typically developing children. We hypothesized that elevated beta oscillations—previously described in awake EEGs in children with Dup15q syndrome—would persist in sleep, and that NREM sleep rhythms that are highly dependent on GABAergic synaptic transmission would be disrupted. Indeed, we found that sleep physiology is abnormal in Dup15q syndrome, characterized by excessive beta oscillations, reduced spindle density, and reduced or absent SWS. Given the fact that most children with Dup15q syndrome undergo clinical overnight EEGs for epilepsy monitoring, findings from this study could guide broader examination and quantification of sleep parameters and inform modifiable targets of intervention, particularly with pharmacological agents that modulate GABA neurotransmission.

### Abnormal sleep physiology in NDDs

Abnormal sleep rhythms have been reported in several neurodevelopmental and neuropsychiatric conditions. Deficits in sleep spindles and REM sleep have been described and quantified in children with non-syndromic ASD [15, 26, 64–66]. Additionally, genetic syndromes highly penetrant for ASD—including Dup15q, Rett, and Angelman syndromes—have shown some unique and some overlapping features in their sleep physiology. In a retrospective, descriptive clinical overnight EEG study of children with Dup15q syndrome, electrical status epilepticus during sleep (ESES), alpha-delta patterns, and periods of high amplitude paroxysmal fast activity were described in approximately 1/3 of patients [67]. Poorly developed spindles and K-complexes, as well as altered SWS have been shown in Rett syndrome [29, 68, 69] and AS [70, 71].

### NREM sleep micro- and macrostructures in Dup15q syndrome

Changes in spindle activity during development are associated with neural maturation. Ontogenesis of sleep spindles in typically developing children may begin at birth but often starts by 3–9 weeks post-term. Initially, spindles appear in the Rolandic regions, are comb-like in morphology, demonstrate prolonged durations up to approximately 10 s, and often occur asynchronously between the hemispheres with relatively low spindle density [72]. Spindle length, morphology, synchrony and density fluctuate over the first few years of life, becoming bifrontocentrally predominant and synchronous by 2 years of age. From 3 years through early adolescence, spindle density continues to increase [72, 73]. Through these well-established changes with age, spindles have been considered an index of neural maturation [74]. Spindles guard offline information processing by suppressing sensory perception of external noise and external stimuli during sleep [75], with spindle features associated with greater resilience to external perturbation [76] and cognitive abilities [77–80]. In neurotypical individuals, spindle density has been correlated with the ability to learn a given task [72].

In addition to an overall reduction in sleep spindle density in our Dup15q cohort, one 54-month-old child notably demonstrated sleep spindles with comb-like morphology that occurred both synchronously and asynchronously between the hemispheres—a pattern found in typical development during infancy or in developmental disorders associated with dysgenesis of the corpus callosum. Brain structural changes have been reported in postmortem studies of Dup15q syndrome, including abnormal neuronal growth and neuronal migration and altered cytoarchitecture [81, 82], which could directly disrupt cortical, subcortical and hippocampal network connectivity and healthy sleep physiology. A connectivity study on patients with AS demonstrated aberrant thalamocortical anisotropy [83], suggesting that there is precedent for chromosomal imbalances affecting the 15q critical region to alter connectivity of brain structures involved in sleep pathways. Future studies that investigate structural and functional connectivity through magnetic resonance neuroimaging (MRI), ideally with diffusion tensor imaging (DTI) tractography, may guide our understanding of the association between altered brain connectivity and abnormal NREM sleep physiology in Dup15q syndrome.

SWS is considered to be the most restorative sleep stage associated with sleep pressure and sleep quality [84]. Slow oscillations during NREM sleep critically stimulate and synchronize other sleep phenomena. For example, physiological ripples ranging from 80 to 100 Hz in humans arising within the CA1 pyramidal layer of the hippocampus have been shown to coordinate with SWS, and they are implicated in the replay of wake-related hippocampal learning activity. Moreover, about 50% of sleep spindles are time-locked to specific phases of slow oscillations [28], resulting in a cross-frequency phase-amplitude coupling. While the role of spindle-slow oscillation coupling in different sleep stages is still under investigation, it is postulated that the coordinated synchrony between thalamocortical spindles, neocortical slow-wave oscillations and hippocampal ripples is critical for brain communication and plasticity and promotes overall cognitive performance [12, 85].

Converging evidence shows that abnormal spindle density and changes in the amount of slow wave activity during the night are highly associated with cognitive impairment [14–16, 26, 72, 86]. In Dup15q syndrome, significant reduction in spindles necessary for nesting into specific phases of slow oscillations may disrupt the temporal coordination between spindles, slow oscillations and hippocampal ripples and, as a result, alter overall brain network communication and plasticity. Disruptions in NREM sleep parameters, therefore, may not only index sleep fragmentation, but may also contribute to and exacerbate the neurodevelopmental disabilities seen in Dup15q syndrome [18–22, 24–28].

### Beta oscillations in Dup15q syndrome

In children with Dup15q syndrome, the duplicated 15q11.2-13.1 gene region includes several genes critical for GABAergic neurotransmission, including *UBE3A* and three GABA_A_R genes. GABA_A_R agonists and modulators such as benzodiazepines induce patterns of beta oscillations very similar to what is observed in children with Dup15q syndrome [53, 87–91]. Typically, the frequency of neural oscillations is determined by time constants on postsynaptic receptors, with faster time constants yielding faster oscillatory frequencies [92]. Benzodiazepines augment the action of GABA by increasing the frequency of GABA_A_R channel opening and decreasing the frequency of beta oscillations while still increasing overall beta power [93, 94]. While highly speculative, this higher amplitude of beta seen in pharmacological GABA_A_R modulation and in Dup15q syndrome likely reflects shifting of faster oscillations towards the beta frequency range [51]. Although beta oscillations are present to varying degrees in all brain states, persistently elevated beta oscillations may inhibit brain state-dependent modulation of neural activity. Interestingly, the converse finding—increased delta power—is seen in individuals with AS, likely reflective of the loss-of-function mutations in the 15q critical region [95, 96]. In AS, GABA_A_R subunit genes are often deleted along with the causative *UBE3A* loss-of-function mutations affecting GABAergic neurons [97], thus highlighting the complex interplay between the 15q critical genes that contribute to the neurophysiologic manifestations seen in the deletion and duplication syndromes.

### Role of GABAergic neurotransmission in sleep physiology

Sleep is a complex and dynamic physiological process that is classified into distinct stages defined by neural oscillatory patterns that can be identified on EEG (Additional file 1 and Additional file 2). Brain state-specific patterns of neurons and brain state-specific neurotransmitters are either activated or inhibited in order to regulate wakefulness and sleep. The basal forebrain (BF), for instance, consists of cholinergic neurons that are active during wakefulness and REM, as well as a heterogeneous group of GABAergic neurons, some of which are active during wakefulness and REM, and others which are active during NREM sleep only [98]. The latter so-called NREM-ON neurons promote sleep through projections within the BF as well as through direct projections to the cortex [99, 100]. Additionally, levels of cortical GABA in the BF neurons are significantly higher during NREM sleep [101]. GABAergic neurons in the ventral tegmental area regulate GABA neurotransmitter release and inhibit wake-promoting orexin/hypocretin neurons, thereby promoting NREM sleep [102]. Overall, neural pathways engaged in NREM sleep tend to be inhibitory, and therefore most sleep-promoting neuronal populations are GABAergic [103, 104].

In the lateral hypothalamus (LH), GABAergic neurons project to the thalamic reticular nucleus (TRN) where they inhibit local TRN GABAergic neurons. Optogenetic and lesion studies have shown that while activation of LH GABAergic neurons induces transitions from NREM to wakefulness, inhibition promotes NREM sleep and delta oscillations [105, 106]. This TRN-mediated inhibitory mechanism is essential in the generation of synchronous thalamocortical oscillations, sleep spindles, thus giving the TRN its name “sleep spindle pacemaker” [28]. GABAergic neurons located within the medulla, striatum and the hypothalamus are critical for the induction of SWS, which is generated within the thalamocortical system and cortically expressed by high amplitude oscillations occurring at a frequency of 0.5–2.0 Hz. During SWS, excitatory and inhibitory neurons throughout cortical layers engage into periods of depolarized “up” states, and hyperpolarized “down” states, the dynamics of which are regulated by the activation of GABA_B_R [107]. Some hypnotics such as the benzodiazepines, which are ligands of GABA_A_ receptors, suppress SWS [108], while hypnotic GABA_B_ receptor agonists such as gamma hydroxybutyric acid increase SWS and improve sleep efficiency [109–111].

Whereas we describe reduced SWS in children with Dup15q syndrome, children with AS demonstrate a higher percentage of SWS [71], suggesting that chromosomal abnormalities involving the 15q region affect the delicate balance of GABAergic neurotransmission required for NREM sleep. Reduced spindle density and abnormal spindle morphology have also been described in children with AS which are thought to be due to aberrant thalamocortical connectivity resulting from GABAergic dysfunction [112]

To our knowledge there are no studies that have directly examined structure or function of the aforementioned brain regions in Dup15q syndrome, and more detailed functional anatomic investigations remain a necessary area of future study. However, the fact that GABAergic neurotransmission plays an essential role in the initiation, synchronization and maintenance of sleep spindles and SWS, and in the regulation of healthy NREM sleep, does support a plausible mechanism for the altered sleep features quantified in this study.

### Clinical implications

As targeted therapeutics emerge in genetically defined neurodevelopmental disorders, there arises a rather urgent need to identify quantifiable mechanistic electrophysiological biomarkers that can shed light on the etiology of cognitive impairment and also serve as a surrogate endpoint in clinical trials. In Dup15q syndrome, abnormal sleep physiology, likely attributable to pathological variants of the *UBE3A* and GABA_A_R genes, can be quantified as elevated beta oscillations in sleep, combined with changes in spindles and reduced SWS. These changes may impair oscillatory synchronization across brain regions during NREM sleep and affect overall brain network function, and may precede or even exacerbate the profound cognitive deficits and behavioral challenges commonly diagnosed in these children.

## Limitations and future directions

Behavioral sleep measures from parental reporting and neuropsychological testing were not available for the entirety of this cohort. However, these promising data have motivated a larger-scale investigation of sleep EEG and behavioral phenotyping to be able to examine correlations between phenotype and electrophysiology. A larger cohort also would allow for the examination of the role of epilepsy, including severity of seizures, anti-epileptic medications, and developmental age that may uniquely impact sleep physiology and contribute to heterogeneity in clinical symptomatology. Moreover, because we relied heavily on clinical EEG quantification, formal sleep staging as would have been performed with PSGs was not possible. However, clinical PSGs are challenging to collect in this population and therefore, we may need to rely on routine overnight EEG recordings as a proxy for sleep monitoring. In fact, the ability to quantify these biomarkers in clinical EEG opens the door for larger-scale studies in syndromic NDDs, where epilepsy is highly penetrant, as individuals with epilepsy undergo routine EEG monitoring on a regular basis. Leveraging access to these clinical data reduces the cost, time and stress of bringing children to research centers for additional data collection.

We speculated in this paper about mechanisms underlying these abnormal sleep patterns, but future clinical and pre-clinical studies could directly elucidate etiology. Clinically, combined MRI and EEG studies will allow us to examine brain structural abnormalities that may contribute to altered brain network connectivity. Quantification of sleep electrophysiological recordings in pre-clinical models of Dup15q syndrome, particularly those with and without the overexpression of GABA_A_R genes, will directly elucidate the effect of putative genes in the 15q region on altered sleep physiology and may also allow for both behavioral and pharmacological manipulations that could improve sleep and learning mechanisms.

## Conclusions

While research in EEG biomarkers has traditionally focused on oscillatory changes in the EEG during wakefulness, our findings suggest that studying sleep physiology in NDDs may be extremely valuable in helping identify quantitative biomarkers of sleep, behavior and cognitive function. The quantitative methods used in this study could be applied to other NDDs. While sleep spindles and slow-wave oscillations may be detected by qualitative measurements, subtle features may be difficult to capture clinically. Quantitative semi-automated measures can identify differences in sleep physiology and help identify biomarkers across syndromic NDDs. Insights gained from this study deepen our understanding of the pathophysiology in Dup15q syndrome and may lay the foundation for studies that investigate the relationship between sleep and cognition, with the ultimate goal of testing specific therapeutics to alter sleep physiology and potentially enhance cognitive development and overall clinical outcomes in children with Dup15q syndrome.

## Supplementary Information

**Additional file 1.** Overview of sleep stages. A sample hypnogram depicting the different sleep stages over the course of one night and representative EEG frequencies during each sleep stage.

**Additional file 2.** Non-REM sleep stages (N1 to N3) in neurotypical children. Representative 9-second traces of continuous sleep EEG recording from neurotypical children depicting vertex waves (field highlighted by blue rectangle) during stage N1 in a 167-month-old patient (**A**), K-complexes (broad field highlighted by blue rectangles) during stage N2 juxtaposed with sleep spindles (arrow) in a 167-month-old patient (**B**), immature synchronous bilateral (arrow) and asynchronous spindles in the right hemisphere alone (hollow arrow) in a 7-month-old patient (**C**) and slow-wave sleep during stage N3 in a 167-month-old patient (**D**).

**Additional file 3.** Beta oscillations in Dup15q syndrome. Time-frequency plots derived from 7 hours of overnight sleep EEG from a 18-month-old representative participant (**A**) and an older, 105-month-old participant (**B**) with Dup15q syndrome.

## Data Availability

EEG data from study participants are available from the corresponding author on reasonable request.

## Abbreviations

AASM: American Academy of Sleep Medicine
ADHD: Attention deficit-hyperactivity disorder
ASD: Autism spectrum disorder
ASR: Artifact subspace reconstruction
BCH: Boston Children’s Hospital
Dup15q syndrome: Duplication 15q11.2-q13.1 syndrome
EEG: Electroencephalogram
GABA_A_R: Gamma-aminobutyric acid type A receptor
IC: Independent component
ID: Intellectual disability
IDDRC: Intellectual Disability and Development Research Center
LH: Lateral hypothalamus
MRI: Magnetic resonance neuroimaging
NDD: Neurodevelopmental disorders
NREM: Non-rapid eye movement
NT: Neurotypical
PSG: Polysomnogram
REM: Rapid eye movement
SWS: Slow-wave sleep
TRN: Thalamic reticular nucleus
UCLA: University of California, Los Angeles
