# Supplementary figures and images for: Abnormal sleep physiology in children with 15q11.2-13.1 duplication (Dup15q) syndrome
Source: Mol Autism. 2021 Aug 3;12:54. doi: 10.1186/s13229-021-00460-8 (PMC8336244; doi:10.1186/s13229-021-00460-8)

## Sleep stages

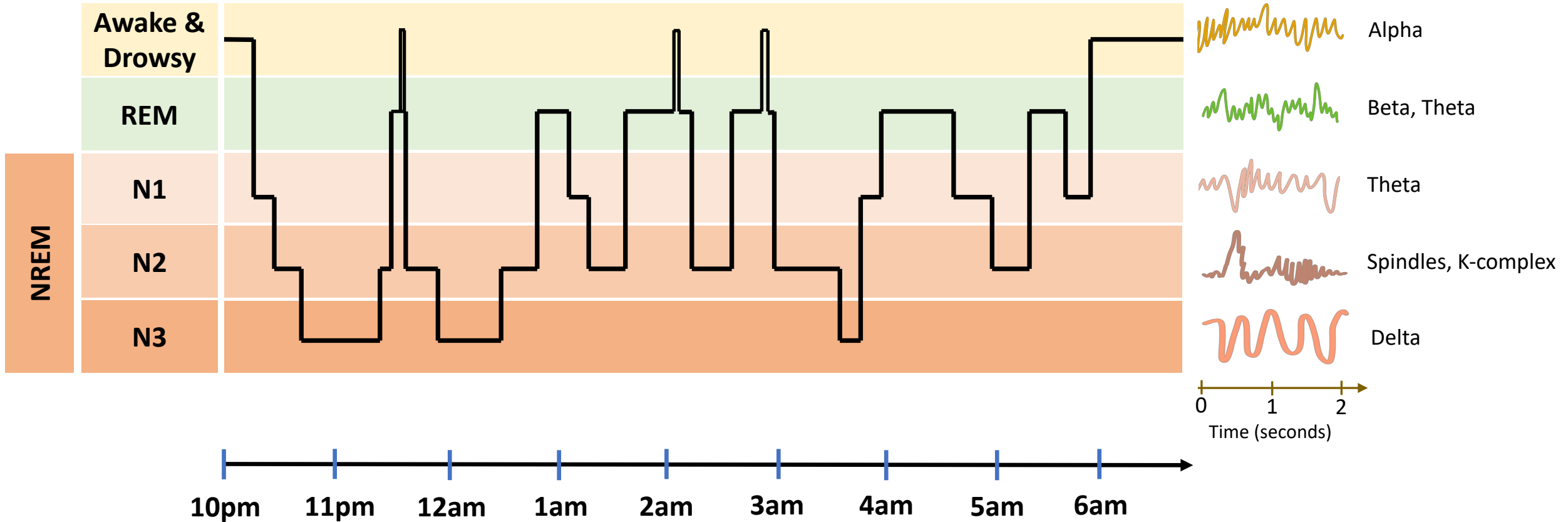

Supplement: Supplementary file 1 — Additional file 1. Overview of sleep stages. A sample hypnogram depicting the different sleep stages over the course of one night and representative EEG frequencies during each sleep stage. [file 13229_2021_460_MOESM1_ESM.pdf]

**A**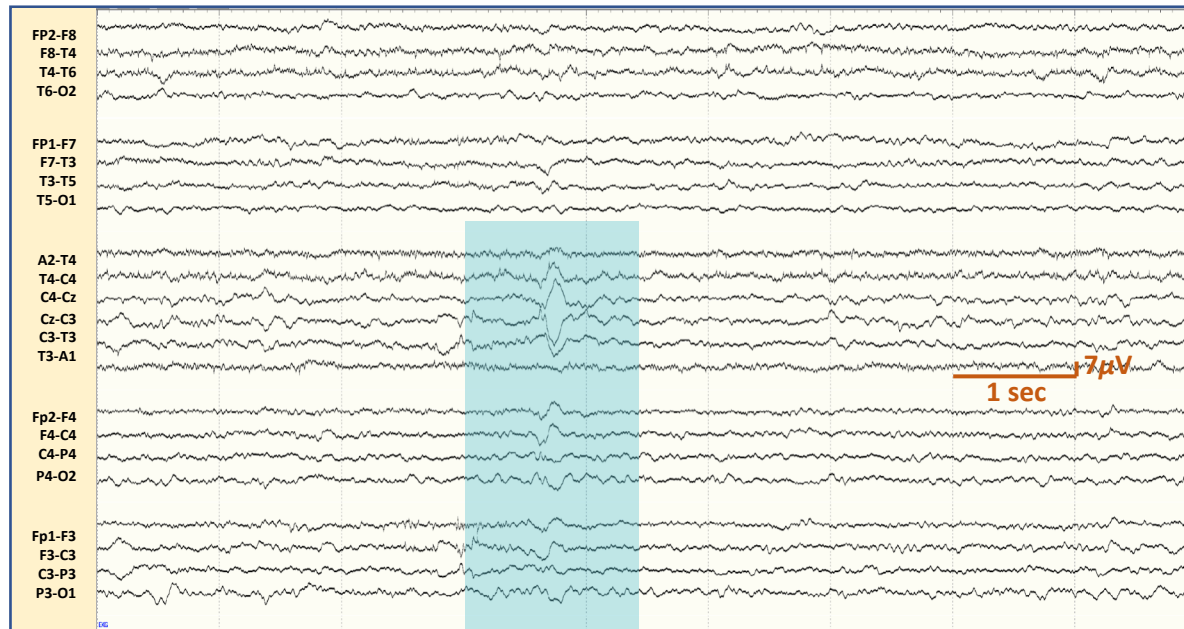**B**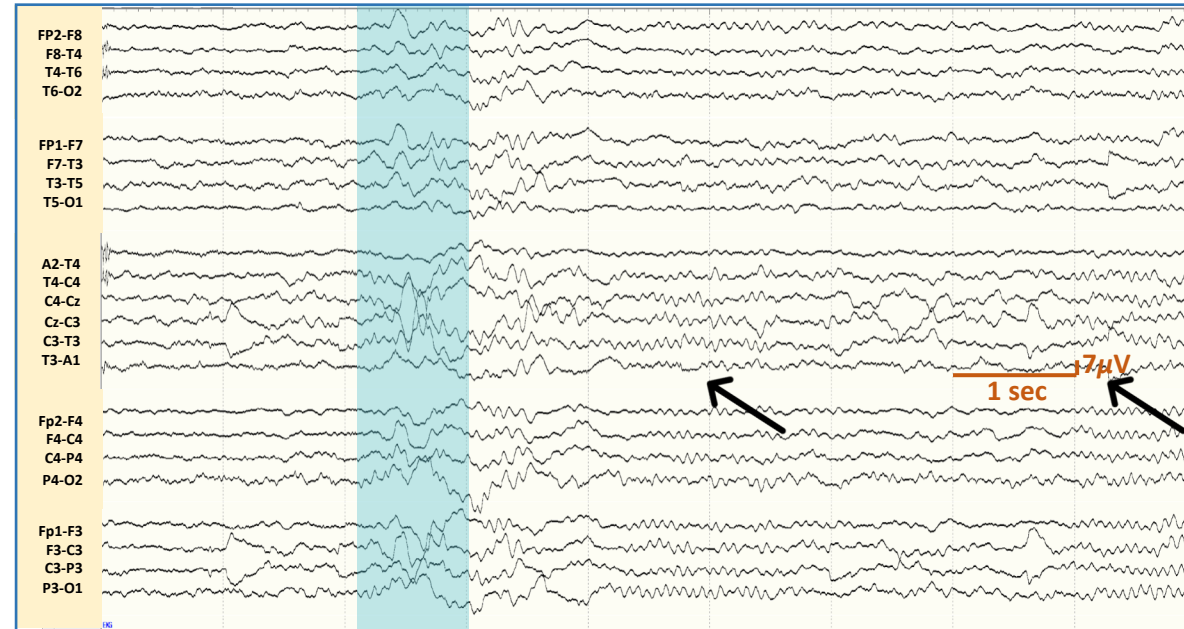**C**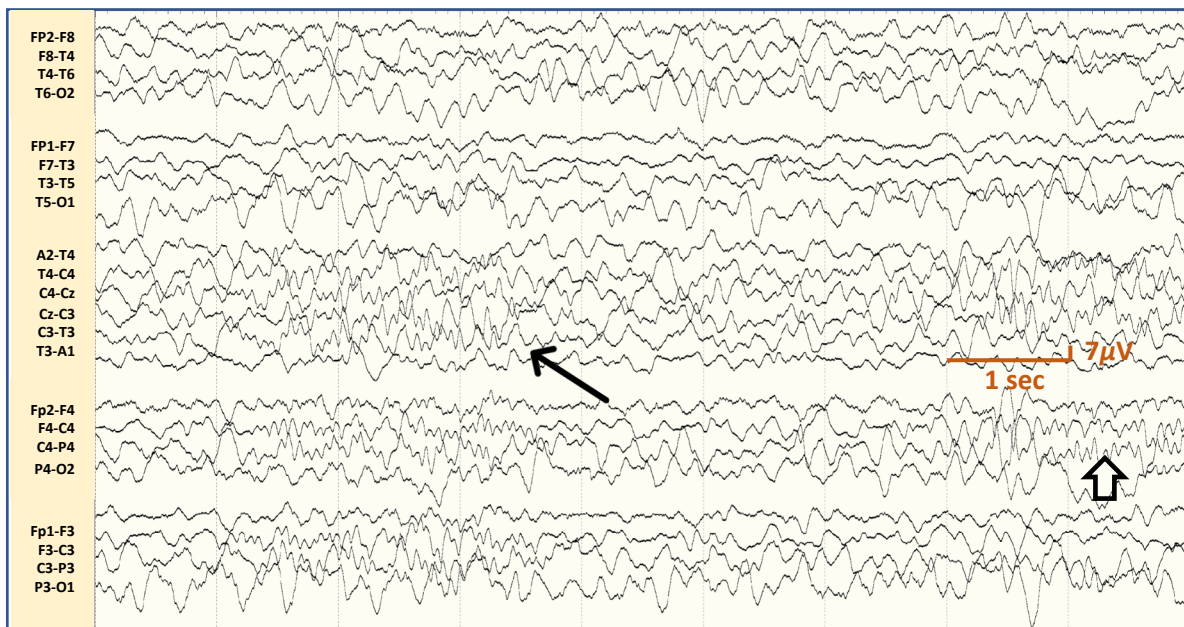**D**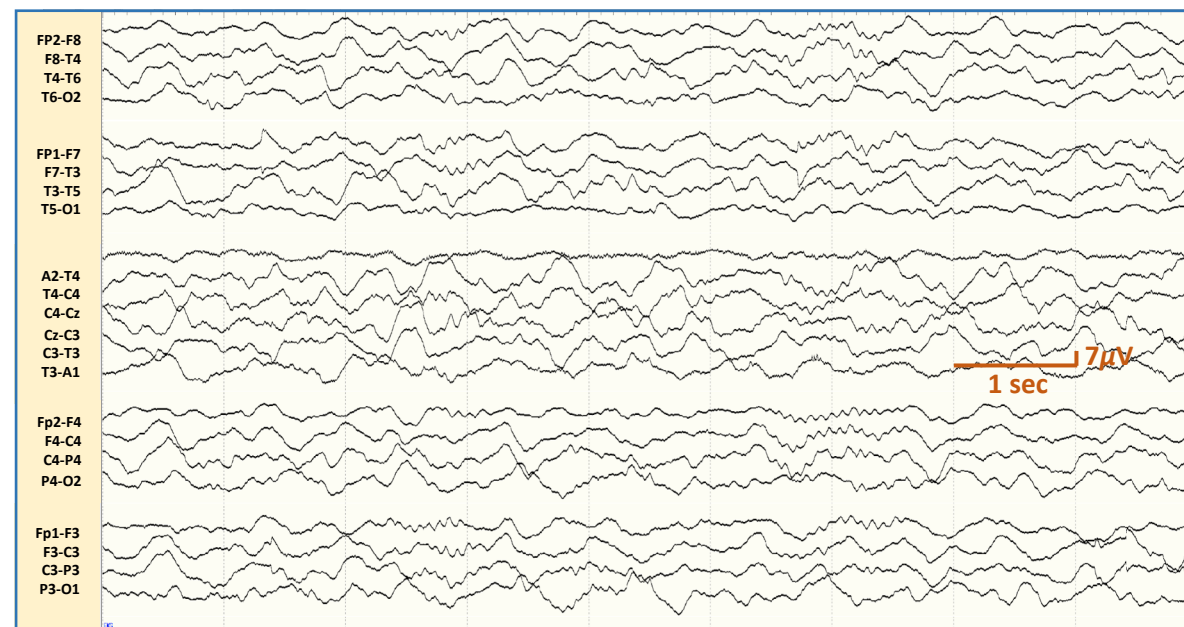

Supplement: Supplementary file 2 — Additional file 2. Non-REM sleep stages (N1 to N3) in neurotypical children. Representative 9-second traces of continuous sleep EEG recording from neurotypical children depicting vertex waves (field highlighted by blue rectangle) during stage N1 in a 167-month-old patient (A), K-complexes (broad field highlighted by blue rectangles) during stage N2 juxtaposed with sleep spindles (arrow) in a 167-month-old patient (B), immature synchronous bilateral (arrow) and asynchronous spindles in the right hemisphere alone (hollow arrow) in a 7-month-old patient (C) and slow-wave sleep during stage N3 in a 167-month-old patient (D). [file 13229_2021_460_MOESM2_ESM.pdf]

**A**

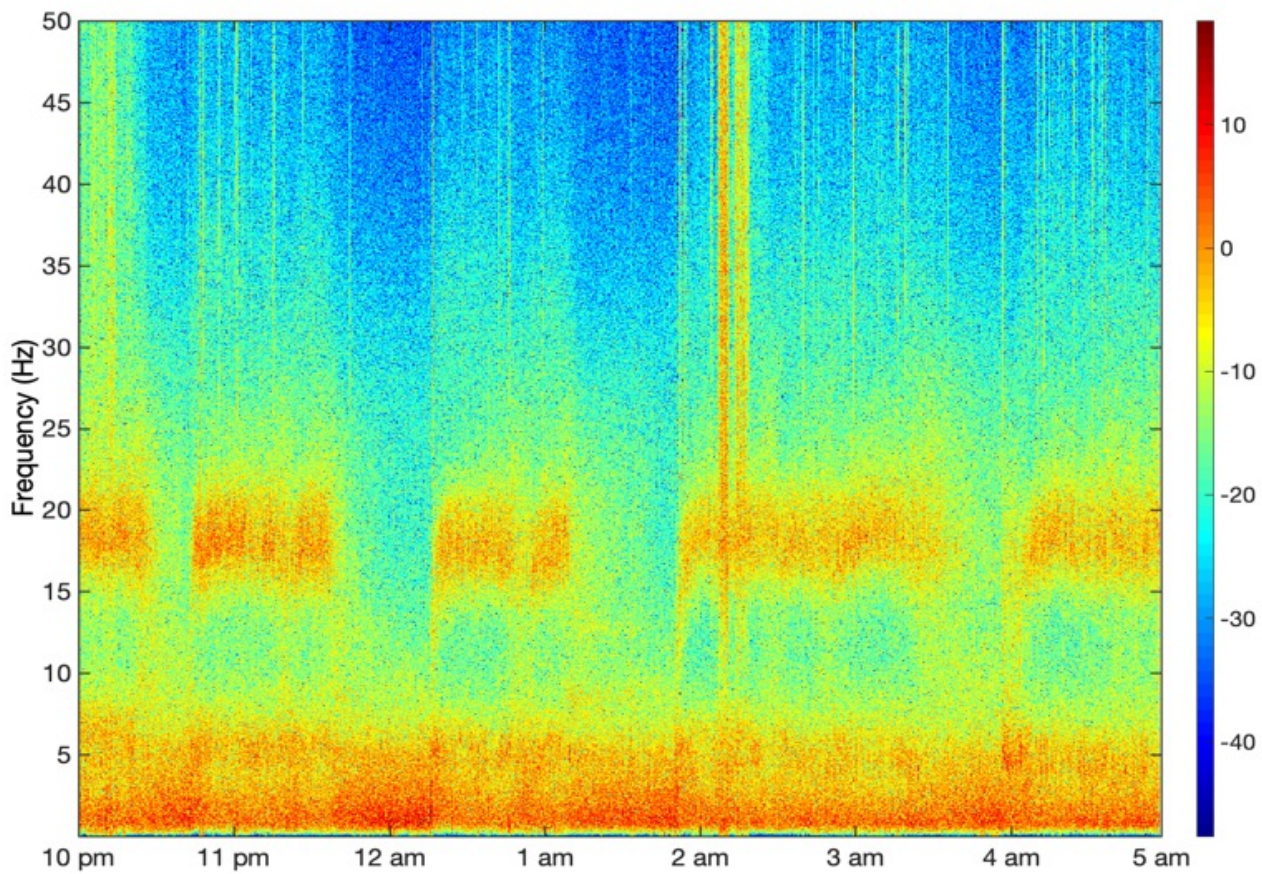

**B**

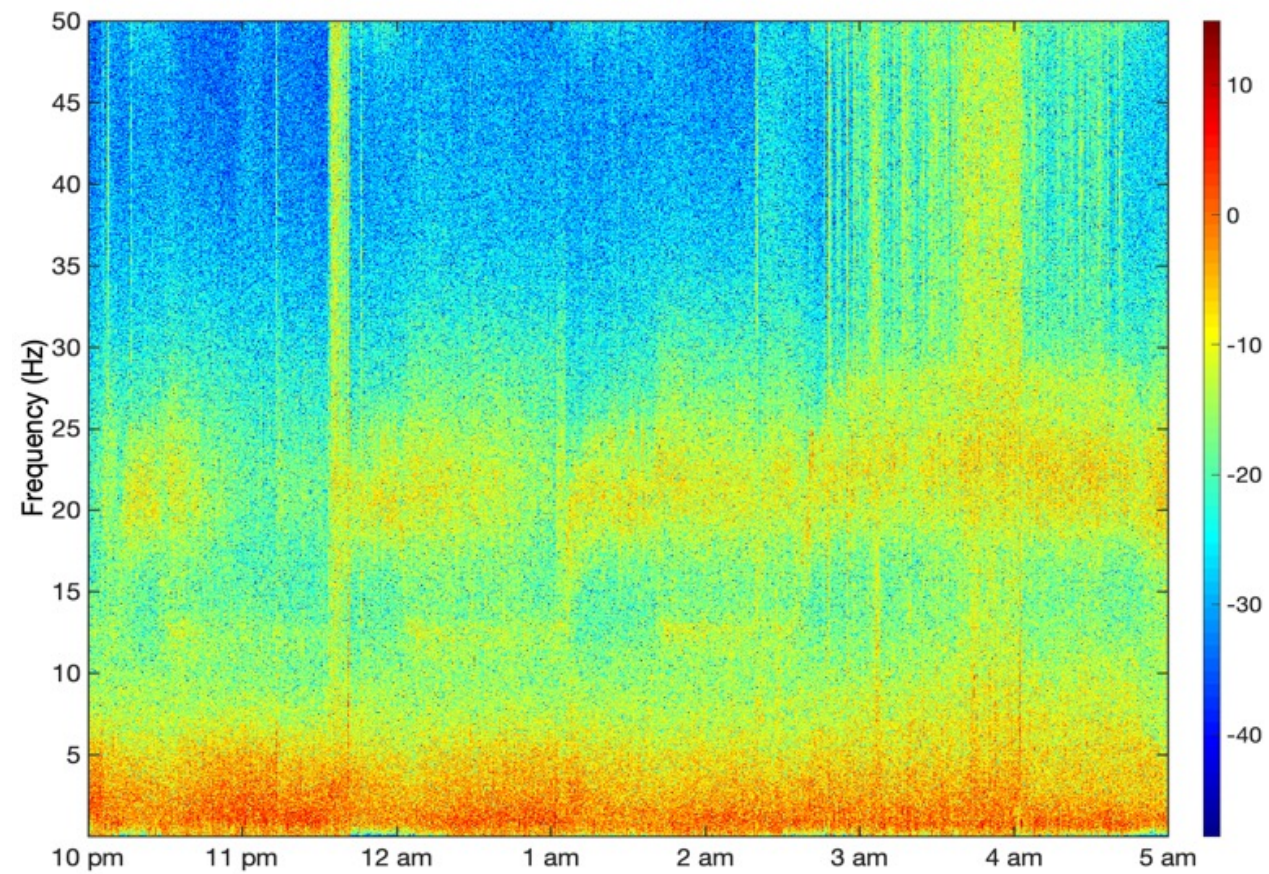

Supplement: Supplementary file 3 — Additional file 3. Beta oscillations in Dup15q syndrome. Time-frequency plots derived from 7 hours of overnight sleep EEG from a 18-month-old representative participant (A) and an older, 105-month-old participant (B) with Dup15q syndrome. [file 13229_2021_460_MOESM3_ESM.pdf]
